# Copy number variants and rasopathies: germline *KRAS* duplication in a patient with syndrome including pigmentation abnormalities

**DOI:** 10.1186/s13023-016-0479-y

**Published:** 2016-07-22

**Authors:** Brigitte Gilbert-Dussardier, Audrey Briand-Suleau, Ingrid Laurendeau, Frédéric Bilan, Hélène Cavé, Alain Verloes, Michel Vidaud, Dominique Vidaud, Eric Pasmant

**Affiliations:** Service de Génétique, C.H.U. de Poitiers, Centre de Référence Anomalies du Développement Ouest, Poitiers, France; EA 3808 Université de Poitiers, Poitiers, France; Service de Génétique et Biologie Moléculaires, Hôpital Cochin, Assistance Publique-Hôpitaux de Paris (AP-HP), Bâtiment Jean Dausset, 3ème étage, 27 rue du Faubourg Saint Jacques, Paris, France; EA7331, Université Paris Descartes, Sorbonne Paris Cité, Faculté de Pharmacie, Paris, France; Assistance Publique des Hôpitaux de Paris (AP-HP), Hôpital Robert Debré, Département de Génétique, INSERM UMR_S1131, Institut Universitaire d’Hématologie, Université Paris Diderot, Sorbonne-Paris-Cité, Paris, France; Assistance Publique des Hôpitaux de Paris (AP-HP), Hôpital Robert Debré, Département de Génétique, INSERM UMR 1141, Université Paris Diderot, Sorbonne-Paris-Cité, Paris, France

**Keywords:** 12p duplication, Café-au-lait spots, CNV, KRAS, Rasopathies

## Abstract

RAS/MAPK pathway germline mutations were described in Rasopathies, a class of rare genetic syndromes combining facial abnormalities, heart defects, short stature, skin and genital abnormalities, and mental retardation. The majority of the mutations identified in the Rasopathies are point mutations which increase RAS/MAPK pathway signaling. Duplications encompassing RAS/MAPK pathway genes (*PTPN11*, *RAF1*, *MEK2*, or *SHOC2*) were more rarely described. Here we report, a syndromic familial case of a 12p duplication encompassing the dosage sensitive gene *KRAS*, whose phenotype overlapped with rasopathies. The patient was referred because of a history of mild learning disabilities, small size, facial dysmorphy, and pigmentation abnormalities (café-au-lait and achromic spots, and axillar lentigines). This phenotype was reminiscent of rasopathies. No mutation was identified in the most common genes associated with Noonan, cardio-facio-cutaneous, Legius, and Costello syndromes, as well as neurofibromatosis type 1. The patient constitutional DNA exhibited a ~10.5 Mb duplication at 12p, including the *KRAS* gene. The index case’s mother carried the same chromosome abnormality and also showed development delay with short stature, and numerous café-au-lait spots. Duplication of the *KRAS* gene may participate in the propositus phenotype, in particular of the specific pigmentation abnormalities. Array-CGH or some other assessment of gene/exon CNVs of RAS/MAPK pathway genes should be considered in the evaluation of individuals with rasopathies.

**Electronic supplementary material:**

The online version of this article (doi:10.1186/s13023-016-0479-y) contains supplementary material, which is available to authorized users.

## Letter to the editor

Rasopathies are a class of genetic syndromes caused by germline mutations in the RAS/mitogen-activated protein kinase (RAS/MAPK) cascade [1], better known for its role in growth factor and cytokine signalling and cancer pathogenesis [2]. Individuals with these syndromes typically present with some combination of facial abnormalities, heart defects, and short stature, although skin and genital abnormalities as well as mental retardation are also common. Germline mutations of genes encoding components of RAS/MAPK pathway have been described in Noonan (NS; OMIM 163950), cardio-facio-cutaneous (CFC; OMIM 115150), Legius (LS; OMIM 611431), and Costello (CS; OMIM 218040) syndromes, capillary malformation and arteriovenous malformation (OMIM 608354) and neurofibromatosis type 1 (NF1; OMIM 162200). The majority of the mutations identified in the rasopathies are mutations which increase RAS/MAPK pathway signaling, many of which are missense mutations [3]. Whole gene deletions have also been reported in patients with *NF1* [4] and duplications encompassing other RAS/MAPK pathway genes (*PTPN11*, *RAF1*, *MEK2*, or *SHOC2*) were more rarely described [5–8]. However, it is sometimes difficult to conclude that an altered RAS/MAPK pathway gene copy number variation (CNV) is critical for the associated phenotype. Here we report, to the best of our knowledge, the first case of a syndromic familial case of a large 12p duplication encompassing the dosage sensitive gene *KRAS*, whose phenotype overlapped with RASopathies.

**Fig. 1:**
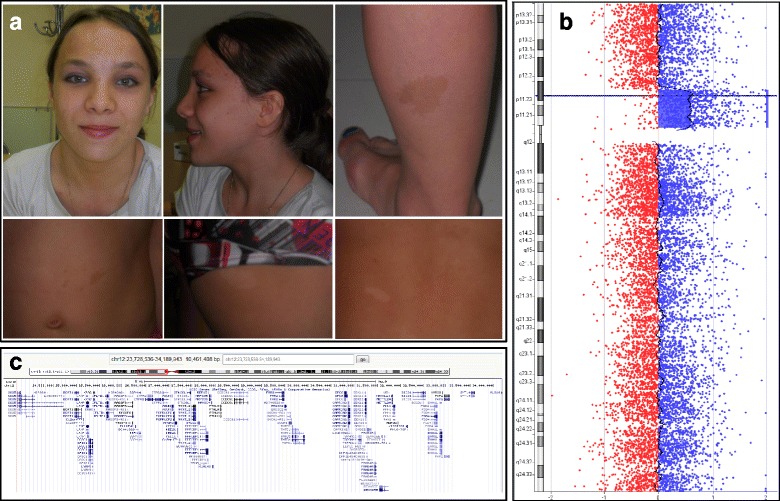
**a** Café-au-lait spots and achromic macules view of the propositus at 14 years of age. Written parental consent was obtained to publish the photograph. **b** Array-CGH profile of the chromosome 12 duplication. **c** Schematic presentation of the 10.4 Mb duplication on chromosome 12 found in the propositus is shown here. The duplication includes *KRAS* as well as multiple other genes (generated with the help of the UCSC Genome Browser at http://genome.ucsc.edu)

We report a patient who was evaluated in our clinic at age 12 and 17 years because of a history of mild learning disabilities (late of 2 years at school), small size (1.35 m as adult = −4 SD), and pigmentation abnormalities: nine café-au-lait spots all over body (the biggest one 3 cm of size), 14 achromic spots, and axillar lentigines (Fig. 1a). We did not observe any evidence for spatial relationship between the café-au-lait spots and the achromic macules. Facial dysmorphy was also noticed, including long face with a broad front and a large philtrum. Bones X rays were normal. She was a premature baby (birth at 27 weeks of pregnancy with birth weight of 1090 g) and had an interauricular communication which improved spontaneously. Her mother had the same phenotype with small size (1.25 m), and coarse face. She died at age 51 years of an unknown cause. This phenotype was reminiscent of RASopathies, among which neurofibromatosis type 1 (NF1), Legius syndrome, cardio-facio-cutaneous (CFC) syndrome, and Noonan syndrome represent prototypic entities [9]. The study was approved by the local ethics committee. Informed consents to participate and to publish were obtained from the patient and her parents. High-molecular-weight DNA was prepared by standard proteinase K digestion followed by phenol-chloroform extraction from whole-blood leukocytes.

The most common genes associated with Noonan, cardio-facio-cutaneous, Legius, and Costello syndromes, as well as neurofibromatosis type 1 were sequenced in the propositus. The coding exons sequencing of the *NRAS*, *PTPN11*, *RAF1*, *SHOC2*, *SOS1*, *SOS2*, *RIT1*, *RASA2*, *LZTR1*, *RRAS*, *BRAF*, *KRAS*, *MAP2K1*, *MAPK2K2*, *NF1*, and *HRAS* genes was performed using targeted-capture next generation sequencing (NGS) as previously described [10]. This genetic screen sequencing identified no mutation.

Genome-wide array-CGH was performed as previously described [11] to identify potential genetic rearrangements. Patient DNA (labelled with Cy5-dUTP) was hybridized on Agilent whole human genome 244 K microarrays (Agilent Technologies) using a pool of genomic constitutional DNAs (leukocytes DNA labelled with Cy3-dUTP) from non-affected individuals as reference. Array was scanned with an Agilent DNA microarray scanner (G2565BA). Log2 ratios were determined with Agilent Feature Extraction software. Results were visualized and analysed with Agilent’s Genomic Workbench 5.0 software. The patient constitutional DNA exhibited a ~10.5 Mb large duplication at 12p (Fig. 1b, c), including 49 protein coding genes, two microRNA genes, and one long non coding RNA gene (Additional file 1: Table S1). The patient’s mother carried the same chromosome abnormality (karyotype: dup(12) (p12.1p11.1)) and also showed development delay with short stature, and numerous café-au-lait spots that were not distinguishable from those of NF1 and Legius syndrome. The duplication observed in the propositus included the *KRAS* gene.

RASopathy-associated constitutional activating mutations in *KRAS* lead to increase in RAS signalling. These mutations are responsible for less than 5 % of *PTPN11* mutation negative Noonan patients or of patients with CFC [9, 12]. The possibility that CNVs encompassing dosage sensitive genes can lead to inherited or sporadic diseases from *de novo* rearrangements was previously discussed [13]. Authors questioned if the increase in the expression of a functionally normal signalling component can mimic the effects of a hyperactive mutant protein. Contribution of CNVs to phenotype can be complex, and interpretation is frequently complicated by the size and type of chromosomal rearrangements, and epigenetic regulation. Whole gene duplication may lead to a weaker increased protein expression than oncogenic activating mutation actually found in *BRAF* or *KRAS* genes. However, although many of the activating mutations are similar to activating somatic mutations seen in cancer, on the whole, they tend to be not as strongly activating in rasopathies. For example, the most common oncogenic *BRAF* mutation, p.Val600Glu, does not occur in CFC syndrome and the specific *KRAS* mutations associated with Noonan syndrome are not the same as the known recurrent somatic mutations associated with cancer. It is likely that the strongly activating oncogenic mutations cannot be tolerated as constitutional mutations [14].

Rasopathy-specific phenotypic traits associated were sometime lacking in previous reported *PTPN11*, *MAP2K2*, or *RAF1* constitutional duplications [6, 7]. Our observation suggests that duplication of the *KRAS* gene may participate in the propositus phenotype, in particular of the specific pigmentation abnormalities. The RAS/MAPK pathway was identified as crucial for controlling pigmentation [15] and some perturbation in the RAS/MAPK cascade can result in multiple café-au-lait spots, although the exact mechanism remains to be elucidated. Café-au-lait macules are a key diagnostic phenotype of rasopathies: they are the most common first sign of NF1 (and also of the rare Legius syndrome) and they are present in 95 % of NF1 patients by the age of 1 year [16–18]. We conclude that our observation suggests that duplication of the region containing *KRAS* may partly result in the observed syndrome phenotype. Array-CGH or some other assessment of gene/exon CNVs of RAS/MAPK pathway genes should be considered in the evaluation of individuals with rasopathies with no point mutation identified by sequencing.

## Abbreviations

array-CGH, array-comparative genomic hybridization; CNV, copy number variation; CFCS, cardio-facio-cutaneous syndrome; CS, Costello syndrome; LS, Legius syndrome; NF1, neurofibromatosis type 1; NS, Noonan syndrome; OMIM, Online Mendelian Inheritance in Man; RAS/MAPK, RAS/mitogen-activated protein kinase

## Additional file

Additional file 1: Table S1. Gene content of the ~10.5-Mb chromosome 12p duplication between nt 23,728,536 and nt 34,189,943 (NCBI Build hg19), including 49 protein coding genes, two microRNA genes, and one long non coding RNA gene. (DOCX 24 kb)
