# Supplementary material for: Copy number variants and rasopathies: germline KRAS duplication in a patient with syndrome including pigmentation abnormalities
Source: Orphanet J Rare Dis. 2016 Jul 22;11:101. doi: 10.1186/s13023-016-0479-y (PMC4957908; doi:10.1186/s13023-016-0479-y)
Supplement: Additional file 1: Table S1. — Gene content of the ~10.5-Mb chromosome 12p duplication between nt 23,728,536 and nt 34,189,943 (NCBI Build hg19), including 49 protein coding genes, two microRNA genes, and one long non coding RNA gene. (DOCX 24 kb) [file 13023_2016_479_MOESM1_ESM.docx]

**Table S1.** Gene content of the ~10.5-Mb chromosome 12p duplication between nt 23,728,536 and nt 34,189,943 (NCBI Build hg19), including 49 protein coding genes, two microRNA genes, and one long non coding RNA gene.

| **NCBI Gene ID** | **HGNC symbol** | **MIM Gene Accession** | **MIM Gene Description** | **Gene End (bp)** | **Gene Start (bp)** | **MIM Morbid Accession** | **MIM Morbid Description** |
| --- | --- | --- | --- | --- | --- | --- | --- |
| 6660 | *SOX5* | 604975 | Sry-Box 5 | 23,682,437 | 24,715,382 |  |  |
| 586 | *BCAT1* | 113520 | Branched-Chain Aminotransferase 1 | 24,962,957 | 25,102,392 |  | Hyperleucinemia-isoleucinemia or hypervalinemia |
| 3845 | *KRAS* | 190070 | V-Ki-Ras2 Kirsten Rat Sarcoma Viral Oncogene Homolog | 25,358,179 | 25,403,869 | 109800, 114480, 615278, 137215, 211980, 609942, 260350, 163200 | Noonan syndrome, Cardiofaciocutaneous syndrome, Gastric cancer, Lung cancer, Pancreatic carcinoma, Bladder cancer, Breast cancer |
| 79365 | *BHLHE41* | 606200 | Basic helix-loop-helix family, member e41 | 26,272,958 | 26,278,002 | 612975 | Short sleeper |
| 8082 | *SSPN* | 601599 | K-Ras Oncogene-Associated Gene | 26,348,031 | 26,387,709 |  |  |
| 3709 | *ITPR2* | 600144 | Inositol 1,4,5-Triphosphate Receptor, Type 2 | 26,488,284 | 26,986,130 | 160190 | Anhidrosis, isolated, with normal sweat glands |
| 51768 | *TM7SF3* | 605181 | Transmembrane 7 Superfamily, Member 3 | 27,124,502 | 27,167,359 |  |  |
| 9412 | *MED21* | 603800 | Mediator complex subunit 21 | 27,175,454 | 27,183,605 |  |  |
| 387849 | *REP15* | 610848 | Rab15 effector protein | 27,849,427 | 27,850,565 |  |  |
| 60488 | *MRPS35* | 611995 | Mitochondrial ribosomal protein s35 | 27,863,705 | 27,909,236 |  |  |
| 5744 | *PTHLH* | 168470 | Parathyroid Hormone-Like Hormone | 28,111,016 | 28,125,663 | 613382 | Brachydactyly, type E2 |
| 51290 | *ERGIC2* | 612236 | Endoplasmic Reticulum-Golgi Intermediate Compartment Protein 2 | 29,493,578 | 29,534,142 |  |  |
| 10526 | *IPO8* | 605600 | Importin 8 | 30,781,914 | 30,848,928 |  |  |
| 1663 | *DDX11* | 601150 | Chl1-related helicase gene 1 | 31,226,778 | 31,257,732 | 613398 | Warsaw breakage syndrome |
| 636 | *BICD1* | 602204 | Bicaudal D, Drosophila, Homolog Of, 1 | 32,259,712 | 32,536,566 |  |  |
| 121512 | *FGD4* | 611104 | FYVE, RhoGEF, and PH domain-containing protein 4 | 32,552,517 | 32,798,983 | 609311 | Charcot-Marie-Tooth disease, type 4H |
| 10059 | *DNM1L* | 603850 | Dynamin-like protein 1 | 32,832,133 | 32,898,583 | 614388 | Encephalopahty, lethal, due to defective mitochondrial peroxisomal fission |
| 51067 | *YARS2* | 610957 | tyrosyl-tRNA synthetase 2, mitochondrial | 32,878,194 | 32,908,894 | 613561 | Myopathy, lactic acidosis, and sideroblastic anemia 2 |
| 5318 | *PKP2* | 602861 | Plakophilin 2 | 32,943,679 | 33,049,779 | 609040 | Arrhythmogenic right ventricular dysplasia 9 |
| 84920 | *ALG10* | 603313 | ALG10, alpha-1,2-glucosyltransferase | 34,175,215 | 34,181,236 | 613688 | Long QT syndrome, acquired, reduced susceptibility to |
| 100126320 | *MIR920* |  | microRNA 920 | 24,365,355 | 24,365,429 |  |  |
| 144360 | *LINC00477* |  | Long intergenic non-protein coding RNA 477 | 24,719,898 | 24,737,102 |  |  |
| 196415 | *C12orf77* |  | Chromosome 12 open reading frame 77 | 25,146,358 | 25,150,373 |  |  |
| 4033 | *LRMP* | 602003 | Lymphoid-Restricted Membrane Protein | 25,205,180 | 25,261,268 |  |  |
| 55259 | *CASC1* |  | Cancer susceptibility candidate 1 | 25,261,223 | 25,348,096 |  |  |
| 144363 | *LYRM5* |  | LYR motif containing 5 | 25,348,150 | 25,357,949 |  |  |
| 160492 | *LMNTD1* |  | Lamin tail domain containing 1 | 25,629,016 | 25,801,496 |  |  |
| 100422897 | *MIR4302* |  | microRNA 4302 | 26,026,953 | 26,027,012 |  |  |
| 11228 | *RASSF8* | 608231 | Ras association domain family, member 8 | 26,111,963 | 26,232,824 |  |  |
| 55726 | *ASUN* | 615079 | Asunder spermatogenesis regulator | 27,058,111 | 27,091,253 |  |  |
| 26127 | *FGFR1OP2* | 608858 | Fibroblast Growth Factor Receptor 1 Oncogene Partner 2 | 27,091,305 | 27,119,581 |  |  |
| 728858 | *C12orf71* |  | chromosome 12 open reading frame 71 | 27,233,990 | 27,235,455 |  |  |
| 23012 | *STK38L* | 615836 | Serine/Threonine Protein Kinase 38-Like Protein | 27,397,077 | 27,478,891 |  |  |
| 56938 | *ARNTL2* | 614517 | Aryl Hydrocarbon Receptor Nuclear Translocator-Like Protein 2 | 27,485,786 | 27,578,745 |  |  |
| 341346 | *SMCO2* |  | Single-pass membrane protein with coiled-coil domains 2 | 27,619,743 | 27,655,118 |  |  |
| 8496 | *PPFIBP1* | 603141 | Protein-Tyrosine Phosphatase, Receptor-Type, F Polypeptide-Interacting Protein-Binding Protein 1 | 27,677,045 | 27,848,497 |  |  |
| 100287284 | *MANSC4* |  | MANSC domain containing 4 | 27,915,599 | 27,924,209 |  |  |
| 57542 | *KLHL42* |  | Kelch-like family member 42 | 27,933,187 | 27,955,973 |  |  |
| 55297 | *CCDC91* |  | Coiled-Coil Domain Containing 91 | 28,343,360 | 28,703,099 |  |  |
| 55711 | *FAR2* | 616156 | Fatty Acyl CoA Reductase 2 | 29,301,935 | 29,488,548 |  |  |
| 341350 | *OVCH1* |  | ovochymase 1 | 29,580,489 | 29,650,619 |  |  |
| 83857 | *TMTC1* | 615855 | Transmembrane And Tetratricopeptide Repeat Domains-Containing Protein 1 | 29,653,745 | 29,937,691 |  |  |
| 65981 | *CAPRIN2* | 610375 | Caprin family, member 2 | 30,862,485 | 30,907,447 |  |  |
| 441631 | *TSPAN11* |  | Tetraspanin 11 | 31,079,536 | 31,149,537 |  |  |
| 58516 | *FAM60A* | 615027 | Family With Sequence Similarity 60, Member A | 31,433,355 | 31,479,158 |  |  |
| 79857 | *FLJ13224* |  | uncharacterized LOC79857 | 31,477,250 | 31,478,879 |  |  |
| 160518 | *DENND5B* |  | DENN/MADD domain containing 5B | 31,535,157 | 31,743,952 |  |  |
| 254013 | *METTL20* | 615256 | Methyltransferase-like 20 | 31,800,093 | 31,822,015 |  |  |
| 196394 | *AMN1* |  | Antagonist of mitotic exit network 1 homolog | 31,824,071 | 31,882,108 |  |  |
| 440093 | *H3F3C* | 616134 | H3 Histone, Family 3C | 31,944,118 | 31,945,174 |  |  |
| 55196 | *KIAA1551* |  | KIAA1551 | 32,112,353 | 32,146,041 |  |  |
| 341359 | *SYT10* |  | Synaptotagmin X | 33,375,413 | 33,439,819 |  |  |
